# Epigenetic activation of SLC7A11 defines a ferroptosis—immune axis and enables robust DNA methylation-based diagnosis of lung squamous cell carcinoma

**DOI:** 10.7717/peerj.20686

**Published:** 2026-02-12

**Authors:** Hui-Ping Lu, Kesong Nong, Lingling Pang, Yulu Tang, Qi Li, Zhendong Chen, Li Xiao, Liangqin Zhu, Dongming Li, Yiyang Chen, Guoqiang Chen, Jingwen Ling, Jiandi Li, Gang Chen, Yi-Wu Dang

**Affiliations:** 1 Department of Pathology, The First Affiliated Hospital of Guangxi Medical University, Nanning, Guangxi, China; 2 Department of Medical Information Engineering, Guangxi Medical University, Nanning, China

**Keywords:** Lung squamous cell carcinoma, SLC7A11, DNA methylation, Diagnostic model, Immune evasion, Ferroptosis

## Abstract

**Background:**

Lung squamous cell carcinoma (LUSC) currently lacks reliable biomarkers for early diagnosis and precision therapy. While Solute Carrier Family 7 Member 11 (SLC7A11) plays key roles in ferroptosis resistance, redox homeostasis and tumor progression, its epigenetic regulation, diagnostic potential, and immunological functions in LUSC remain poorly understood.

**Methods:**

Multi-omics data from The Cancer Genome Atlas (TCGA), Clinical Proteomic Tumor Analysis Consortium (CPTAC), Gene Expression Omnibus (GEO) and an in-house cohorts of 173 LUSC patients were integrated to characterize SLC7A11 DNA methylation, mRNA, and protein levels. Four methylation probes were utilized to construct diagnostic models, including Generalized Linear Model (GLM), Least Absolute Shrinkage and Selection Operator (LASSO), Random Forest (RF), and Extreme Gradient Boosting (XGB). These models were validated internally (*via* 10-fold cross-validation and bootsrtapping) and externally using the GSE121849 dataset. Model interpretability was examined through SHapley Additive exPlanations (SHAP). Additionally, immune infiltration, pathway enrichment and drug sensitivity analyses were performed to explore ferroptosis-associated and immunity-related mechanisms.

**Results:**

SLC7A11 exhibited LUSC-specific epigenetic activation, characterized by promoter hypomethylation, mRNA upregulation, and protein overexpression across cohorts. The four-probe GLM diagnostic model achieved superior performance (AUC = 0.985 in TCGA; AUC = 1.000 in GSE121849), with SHAP identifying cg02102889 (TSS1500) as the most influential probe. While SLC7A11 expression and methylation were not significantly associated with survival in the overall cohort, high SLC7A11 predicted poorer outcomes in female patients and those with pathologic T3 & T4 stage disease. Mechanistically, SLC7A11-high tumors displayed ferroptosis-resistant and immunosuppressive phenotypes, including increased Programmed Death-Ligand 1 (PD-L1) expression and enrichment of regulatory T cells and M2 macrophages. Drug sensitivity profiling suggested resistance to Reactive Oxygen Species (ROS) inducers and Histone Deacetylase (HDAC) inhibitors, but enhanced sensitivity to recombinant tumor necrosis factor-related apoptosis-inducing ligand (rTRAIL) and 17-Allylamino-17-demethoxygeldanamycin.

**Conclusion:**

SLC7A11 undergoes epigenetic activation in LUSC and enables a robust four-probe, methylation-based diagnostic model. Its expression is linked to ferroptosis resistance, immune evasion, and therapeutic response, supporting SLC7A11 as a promising biomarker for early diagnosis and personalized treatment in LUSC.

## Introduction

Lung cancer remains the leading cause of cancer-related mortality worldwide. Lung squamous cell carcinoma (LUSC), a major histological subtype of non-small cell lung cancer, is frequently diagnosed at advance stages and currently relies largely on invasive histopathological assessment, as reliable non-invasive biomarkers are still lacking. Standard treatments such as chemotherapy and radiotherapy, offer limited benefit and often cause substantial toxicity, underscoring the urgent need for molecular biomarkers to improve early diagnosis and guide individualized therapeutic strategies.

Solute Carrier Family 7 Member 11 (SLC7A11), the light-chain subunit of the cystine/glutamate antiporter system xCT, plays a central role in maintaining intracellular redox homeostasis by mediating cystine import and glutamate export. Dysregulated SLC7A11 upregulation has been reported in multiple cancers, where it enhances antioxidant defenses, promotes ferroptosis resistance, and contributes to immune evasion. SLC7A11 protein stability is further modulated by post-translational regulators such as ENF2 and RNF128, while elevated expression has been linked to resistance to chemotherapeutic agents, including cisplatin, paclitaxel, and poly(ADP-ribose) polymerase (PARP) inhibitors (Hong et al., 2021; Li et al., 2022a; Liu et al., 2024a; Liu et al., 2024b; Ando et al., 2025; Aoyama et al., 2025; Gong et al., 2025; Rong et al., 2025; Sun et al., 2025; Zhang et al., 2025a; Zhang et al., 2025b; Zhu et al., 2025), whereas pharmacological inhibition with small molecules such as erastin and sulfasalazine induces ferroptosis and restores chemosensitivity (Zhen et al., 2025). Moreover, SLC7A11 overexpression can remodel the tumor microenvironment by elevating PD-L1 expression and suppressing antitumor immunity (Liu et al., 2021; Xu et al., 2025b). Collectively, these findings highlight SLC7A11 as a central regulator of ferroptosis, redox balance, and immune escape, and suggest its promise as both a diagnostic biomarker and therapeutic target.

Despite these insights, the epigenetic regulation, diagnostic potential and immunological roles of SLC7A11 in LUSC remain largely unexplored. To fill these gaps, we performed an integrative multi-omics study using The Cancer Genome Atlas (TCGA), Clinical Proteomic Tumor Analysis Consortium (CPTAC), Gene Expression Omnibus (GEO) datasets, and an in-house cohort of 173 patients. We systematically characterized SLC7A11 DNA methylation, transcriptional expression and protein abundance; identified methylation-based diagnostic probes; and constructed interpretable diagnostic models with internal and external validation. We further interrogated the biological and clinical relevance of SLC7A11 through immune infiltration profiling, pathway enrichment, single-cell transcriptomics and pharmacogenomic analyses. Together, our findings provide new mechanistic insights into SLC7A11-driven metabolic and immune dysregulation in LUSC and support its potential as a clinically actionable biomarker.

## Materials & Methods

### Data source and preprocessing

Multi-omics data for pan-cancer, including RNA-seq (STAR-Counts), DNA methylation (Illumina HumanMethylation450K), clinical annotation, and survival information, were obtained from the UCSC Xena browser. The UALCAN portal (Chandrashekar et al., 2022), which integrate TCGA and CPTAC datasets, was used to compare SLC7A11 promoter methylation, mRNA expression, and protein abundance between tumor and normal tissues. Independent microarray datasets were retrieved from GEO, including GSE121849 (external diagnostic cohort) and GSE127465 (single-cell RNA-seq dataset).

Gene expression matrices were annotated using ENSEMBL-to-SYMBOL mapping and normalized to log_2_(TPM+1). DNA methylation levels were represented as β values. Basic preprocessing, including normalization, batch correction (when applicable) and quality control, was performed according to standard pipelines. Ferroptosis-related genes were retrieved from FerrDb v2, and NRF2 pathway gene sets were sourced from MSigDB.

### Patient cohort and immunohistochemistry

Formalin-fixed paraffin-embedded tumor tissues and paired adjacent lung tissues from 173 LUSC patients were collected at the First Affiliated Hospital of Guangxi Medical University between October 2019 and March 2023. Immunohistochemistry (IHC) staining for SLC7A11 was performed using a rabbit polyclonal antibody (Proteintech, Cat No. 26864-1-AP; Wuhan, China; dilution 1:1000). Antigen retrieval was conducted using citrate buffer (10 mM, pH 6.0) under high-pressure for 2.5 min. Phosphate buffered saline (PBS) was used as a negative control, and human lung cancer tissue known to express SLC7A11 (as recommended by manufacturer) served as a positive control. Signal detection was performed using DAB (ZSGB-Bio, Cat No. PV-6000; Beijing, China), followed by hematoxylin counterstaining.

IHC staining scores were evaluated according to previously published criteria (Zhang et al., 2023). Clinical parameters, including sex, pathological TNM stage, Ki-67 index, and histological grade, were collected for stratified analyses. This study were approved by the Institutional Ethics Committee of the First Affiliated Hospital of Guangxi Medical University (Approval No. 2023-S706-01), and written informed consent was obtained from all patients.

### DNA methylation and expression analysis

Differentially methylated probes (DMPs) associated with SLC7A11 were identified in TCGA-LUSC using the limma package, with selection thresholds of adjusted *P*-value < 0.01, —logFC— > 0.1, and B-statistic > 1 as selection criteria. Associations between probe methylation (β values) and SLC7A11 mRNA expression were evalusted using Spearman’s correlations.

### Construction and evaluation of diagnostic models

Four SLC7A11-related DMPs were used to construct methylation-based diagnostic classifiers, including generalized linear model (GLM), random forest (RF), least absolute shrinkage and selection operator (LASSO), and extreme Gradient Boosting (XGB) models. TCGA-LUSC served as the primary training cohort. Model performance was evaluated by the area under the receiver operating characteristic curve (AUC), sensitivity, specificity, and optimal cutoff values (*via* pROC and caret packages). To assess internal robustness and overfitting, we performed 10-fold cross-validation and 1,000-iteration bootstrapping validation within TCGA-LUSC. The GSE121849 cohort was used as an independent external validation set, where the trained models were applied without re-fitting, and AUC values were calculated to assess generalizability. Model interpretability was evaluated using SHapley Additive exPlanations (SHAP) *via* the SHAPforxgboost package, with a focus on contributions of individual CpG probe.

### Functional enrichment and protein–protein interaction analysis

Differential expression analysis between SLC7A11-high and SLC7A11-low groups (stratified by median expression) was performed in TCGA-LUSC. Gene Set Enrichment Analysis (GSEA) was conducted using clusterProfiler and the Xiantao web server (https://www.xiantaozi.com/). Protein–protein interaction (PPI) networks were constructed using STRING (https://string-db.org/) with the minimum required interaction score set to 0.400 (medium confidence).

### Ferroptosis, NRF2 pathway and drug sensitivity analysis

To explore ferroptosis- and NRF2-related mechanism, differentially expressed genes (DEGs; adjusted *P*-value < 0.05 and —log_2_FC— ≥ 2) were intersected with ferroptosis-related genes and NRF2 pathway genes. Co-expression patterns among SLC7A11 and ferroptosis/NRF2-related genes were evaluated using Spearmon’s correlation, and heatmaps were generated *via* Xiantao.

Drug sensitivity analysis was performed using the GSCAlite platform (Liu et al., 2023) (https://guolab.wchscu.cn/GSCA/#/drug), which integrates cell line-based pharmacogenomic data from the Genomics of Drug Sensitivity in Cancer (GDSC) database. The half-maximal inhibitory concentration (IC50) values of 265 small-molecule drugs and gene expression profiles from 860 cancer cell lines were integrated. Pearson correlation analysis was conducted to assess the association between SLC7A11 mRNA expression and drug IC50. Multiple testing correction was applied using the false discovery rate (FDR). GSCALite automatically ranks drug-gene pairs using an integrated score combining correlation magnitude and statistical significance, and visualizes the top-ranked interactions in a bubble plot. Drugs with —correlation— > 0.1 and FDR < 0.05 were considered significant.

### Immune infiltration and immune checkpoint co-expression analysis

Immune cell infiltration in LUSC was estimated using the CIBERSORT algorithm with LM22 reference signature (100 permutations) (Xu et al., 2025a). To provide external validation, CIBERSORT-ABS implemented in TIMER2.0 (Li et al., 2020b) was additionally applied, which generates absolute immune cell scores rather than relative fractions. Association between SLC7A11 expression and immune checkpoint genes (He et al., 2018), including CD274/PD-L1 and other co-inhibitory/co-stimulatory molecules, were assessed using Spearman’s correlation. Heatmaps were drawn with Xiantao. PPI networks among immune-related genes were constructed visualized with STRING.

### Single-cell RNA sequencing analysis

Single-cell RNA sequencing (scRNA-seq) data from GSE127465 (platform: GPL18573) were processed with Seurat pipeline (Heumos et al., 2023). Quality-control filtering retained cells with nFeature_RNA > 200 and mitochondrial gene percentage < 20%. Gene expression was log-normalized, and the top 2,000 variable genes were selected for downstream analysis. Principal component analysis (PCA) was performed, and the top 20 principal components were used for clustering (FindNeighbors and FindClusters with resolution = 0.5). Uniform Manifold Approcimation and Projection (UMAP) was used for dimentionality reduction and visualization.

Cell types were annotated using reported canonical marker genes. Cells were stratified into SLC7A11-high and SLC7A11-low groups according to the median expression. DEGs (adjusted *P*-value < 0.05 and —log_2_FC— > 0.25) between the two groups were identified using the FindMarkers function. Upregulated and downregulated genes were subjected to GSEA using the clusterProfiler package, which was assisted by Xiantao.

### Survival analysis

The associations between SLC7A11 methylation probes and overall survival (OS) were assessed using the MethSurv online platform (Modhukur et al., 2018) (https://biit.cs.ut.ee/methsurv/). In TCGA-LUSC, survival analyses for SLC7A11 expression were conducted using the survival and survminer packages, including univariate Cox proportional hazards regression and Kaplan–Meier survival curves for OS, disease-specific survival (DSS) and progression-free interval (PFI). Subgroup analyses were performed according to sex and pathological T stage.

### Statistic

All statistical analyses were performed in R (version 4.4.0) and Bioconductor framework (version 3.20). Group comparisons were conducted using Student’s *t*-test or Wilcoxon rank-sum test depending on distribution. Multiple testing correction used the Benjamini–Hochberg method. Kaplan–Meier survival curves were compared by log-rank tests. Unless otherwise specified, a two-sided *P*-value < 0.05 was considered statistically significant.

## Results

### SLC7A11 was epigenetically activated in LUSC

Integration of the Cancer Cell Line Encyclopedia, TCGA, and CPTAC datasets showed that SLC7A11 was broadly upregulated across multiple cancer types, displayed moderate-to-high diagnostic performance (AUC > 0.7), and was associated with adverse survival outcomes in specific clinical subgroups (Supplementary Material S1–S6). These patterns suggested an oncogenic role for SLC7A11, but its regulatory mechanisms varied substantially across cancers.

**Figure 1:**
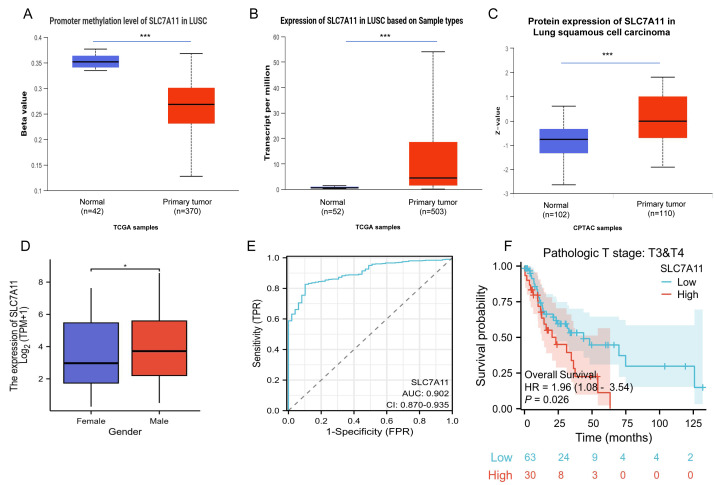
Multi-omics evidence of SLC7A11 activation in LUSC. (A) The promoter methylation levels of SLC7A11 in LUSC *versus* normal tissues. (B) The mRNA expression levels of SLC7A11 in LUSC *versus* normal tissues. (C) The protein expression levels of SLC7A11 in LUSC *versus* normal tissues. (D) Differential mRNA expression of SLC7A11 between male and female patients. (E) Diagnostic ROC curve of SLC7A11 in LUSC. (F) Overall survival of patients with advanced pathologic T stage (T3&T4) according to SLC7A11 expression. * *P* < 0.05, *** *P* < 0.001.

Strikingly, LUSC was the only tumor type exhibiting concordant multi-omics activation, characterized by promoter hypomethylation, mRNA upregulation and protein overexpression. Three promoter methylation probes (cg00361146 at transcription start site (TSS)1500, cg13028471 at TSS200 and cg02102889 at TSS1500) showed significantly lower β values in tumors compared with normal tissues (mean β: 0.268 *vs.* 0.352, *P* = 1.624 × 10^−12^), correspondingly, SLC7A11 mRNA expression was markedly elevated in LUSC (mean log_2_TPM: 4.308 *vs.* 0.508 in normal tissues, *P* < 1 ×10^−12^) and CPTAC data confirmed a consistent increase in protein abundance (mean *Z*-score: 0 *vs.* −0.771 in normals) (Figs. 1A–1C). A significant sex-specific difference was observed, with male patients showing higher SLC7A11 expression than female patients (mean log_2_(TPM+1): 3.928 *vs.* 3.463, *P* = 0.021; Fig. 1D).

Diagnostic evaluation demonstrated excellent discrimination between tumors and normal tissues (AUC = 0.902, 95% CI [0.870–0.935]; Fig. 1E). To further evaluate the generalizability of SLC7A11 as a diagnostic biomarker, we examined whether promoter methylation differed across racial subgroups in the TCGA cohort. Although normal lung tissues exhibited higher methylation levels than tumors, no significant methylation differences were observed among Caucasian, African American, and Asian LUSC patients (Supplementary Material S9). These findings suggest that SLC7A11 hypomethylation is a tumor-driven alteration rather than a race-dependent effect, supporting its potential applicability across diverse populations.

Although SLC7A11 expression was not prognostic for OS, DSS or PFI in the overall TCGA-LUSC cohort (Supplementary Material S6), subgroup analyses demonstrated significantly shorter OS in patients with high expression and advanced pathological T stage (T3 & T4 stage; HR = 1.96, 95% CI [1.08–3.54]; *P* = 0.026; Fig. 1F). In addition, MethSurv analysis identified two CpG probes (cg05373863 and cg24869834) significantly associated with OS (*P* = 0.0028 and 0.048), supporting the prognostic relevance of SLC7A11 methylation (Supplementary Material S6).

Collectively, these findings indicate that SLC7A11 undergoes LUSC-specific epigenetic activation and may serve as a promising biomarker for diagnostic and prognostic stratification.

### Identification of four diagnostic methylation probes

**Figure 2:**
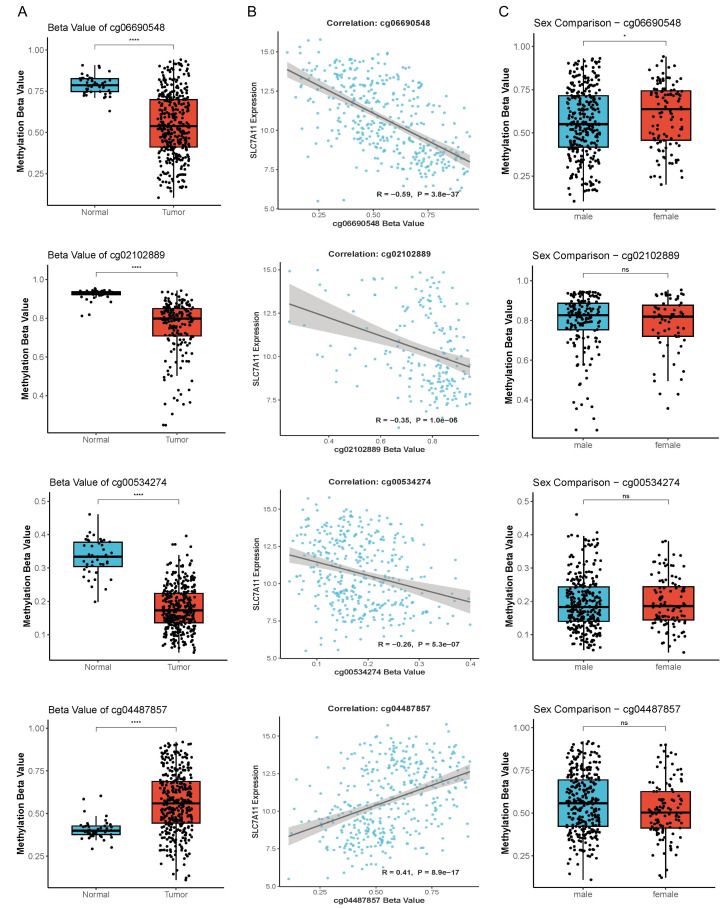
Identification and correlation of SLC7A11 methylation probes. (A) Differential methylation of four CpG probes in normal and tumor tissues. (B) Correlation between probe methylation levels and SLC7A11 m RNA expression. (C) Sex-specific methylation difference of the four probes. * *P* < 0.05, **** *P* < 0.0001.

Because TCGA does not contain proteomic measurements for SLC7A11 and CPTAC does not provide matched methylation and proteomic measurements for the same patients, the methylation-mRNA relationship was assessed using TCGA, which offers fully matched DNA methylation and RNA-seq profiles. Based on TCGA-LUSC Illumina 450K methylation data, four differentially methylated probes (cg00534274, cg02102889, cg04487857, and cg06690548) were identified as potential diagnostic markers (Table 1). Among them, three probes (cg00534274, cg02102889, and cg06690548) were significantly hypomethylated in tumors and negatively correlated with SLC7A11 expression (*R* =  − 0.26 to −0.59, all *P* < 0.001), whereas cg04487857 was hypermethylated (*P* < 0.001) and positively correlated with mRNA levels (*R* = 0.41, *P* < 0.001) (Figs. 2A–2B). Probe annotation revealed functional relevance: cg00534274 mapped to the first exon and 5′ untranslated region (5′-UTR), cg02102889 to the TSS1500 region, both cg06690548 and cg04487857 to the gene body (Table 1), implicating potential roles in transcriptional initiation and elongation. Sex-stratified analyses identified significantly lower methylation of cg06690548 in male patients (Fig. 2C). Together, these results highlight promoter and gene-body methylation as key contributors to SLC7A11 epigenetic activation.

**Table 1:** Functional annotation of four key SLC7A11-associated CpG probes.

Probes ID	UCSC_RefGene_Group	logFC	AveExpr	*t*	*P*.Value	*adj.P*.Val	*β*.Value
cg00534274	1stExon;5′UTR	−0.153	0.195	−14.928	1.501 ×10^−40^	1.276 ×10^−39^	81.258
cg06690548	Body	−0.239	0.573	−8.145	4.585 ×10^−15^	1.949 ×10^−14^	23.037
cg02102889	TSS1500	−0.170	0.787	−7.579	9.013 ×10^−13^	3.064 ×10^−12^	17.956
cg04487857	Body	0.155	0.548	5.735	1.886 ×10^−08^	5.343 ×10^−08^	8.104

### Construction and validation of a methylation-based diagnostic model

**Figure 3:**
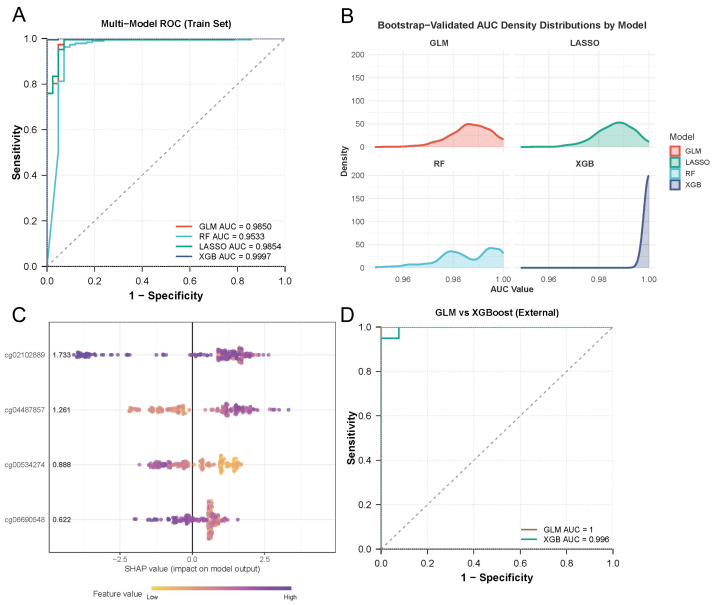
Diagnostic modeling and external validation. (A) ROC curves of GLM, LASSO, RF, and XGB classifiers in the training set (TCGA). (B) Bootstrap-validated AUC distribution for each model. (C) SHAP values showing probe importance in the diagnostic model. (D) External validation performance of GLM and XGB models in validated set (GSE121849).

The four CpG probes were incorporated into GLM, RF, LASSO, and XGB classifiers. TCGA-LUSC cohort was used exclusively as the training cohort, while GSE121849 served as an independent external validation set, thereby avoiding information leakage between cohorts. In the training cohort, the GLM model achieved the best diagnostic accuracy (AUC = 0.985), outperforming RF (AUC = 0.963), LASSO (AUC = 0.948), and XGB (AUC = 0.972) (Fig. 3A; Table 2). To minimize potential overfitting, internal validation demonstrated strong robustness, confirm by 1,000 bootstrap iterations (mean AUC = 0.987 ± 0.008) and 10-fold cross-validation (mean AUC = 0.982) (Fig. 3B; Table 2). Model interpretability analysis using SHAP revealed that cg02102889 (TSS1500) was the most influential feature (mean SHAP value = 1.733), followed by cg00534274, cg06690548, and cg04487857 analysis of XGB model identifier (Fig. 3C). External validation was performed in an independent GEO dataset (GSE121849), consisting of 20 LUSC tumors and 13 matched normal tissues. The GLM classifier achieved an AUC of 1.000 with 100% sensitivity and 100%cpecificity, whereas XGB achieved an AUC of 0.996 (Fig. 3D). These results confirmed the robustness, interpretability, and reproducibility of the methylation-based diagnostic models.

### In-house IHC validation confirmed SLC7A11 overexpression and revealed sex-specific differences

**Figure 4:**
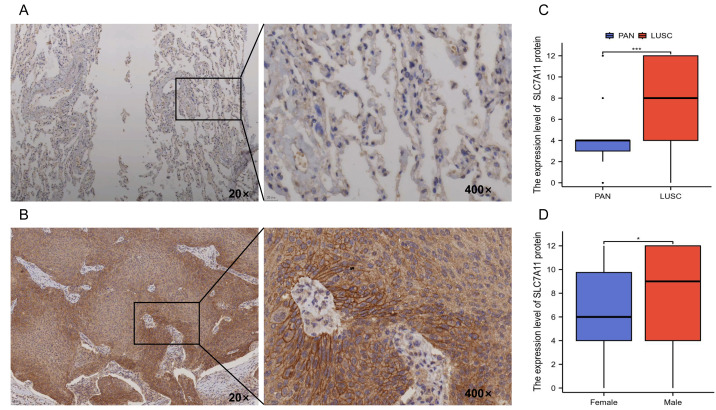
Immunohistochemical analysis of SLC7A11 protein expression in the in-house LUSC cohort. (A) Representative staining of paired adjacent normal (PAN) lung tissues. (B) Representative staining of LUSC tissues. (C) Comparison of SLC7A11 protein expression between PAN and LUSC tissues. (D) Comparison of SLC7A11 protein expression between female and male patients. * *P* < 0.05; *** *P* < 0.001.

To validate the multi-omics findings, we performed IHC on an independent in-house 173-patient paired LUSC cohort (Supplementary Material S7). Consistent with CPTAC proteomic data, SLC7A11 protein was strongly expressed in malignant squamous cells with both plasma membrane and cytoplasmic staining localization. Protein levels were significantly higher in tumor than in adjacent normal tissues (8.057 ± 3.793 *vs.* 4.343 ± 2.650; *P* = 9.12 × 10^−20^) (Figs. 4A–4C). Consistent with TCGA-LUSC transcriptomic profiling, male patients exhibited higher protein levels than female patients (8.343 ± 3.713 *vs.* 6.6429 ± 4.002; *P* = 0.035) (Fig. 4D), in agreement with sex-associated epigenetic changes observed in methylation probes (Fig. 2C). However, no significant association were found between SLC7A11 expression and age, pathological T/N/M stage, Ki-67 index, or pathological grade (all *P* > 0.05), indicating that SLC7A11 dysregulation was not simply driven by tumor burden or proliferation (Supplementary Materials S8, S13). Together, these findings confirm consistent SLC7A11 overexpressing across transcriptomic, proteomic, and histological levels, with a reproducible sex-associated difference.

**Table 2:** Performance metrics of diagnostic models in training and validation cohorts.

Model	Train *AUC*	Train *Sensitivity*	Train *Specificity*	Boot *AUC*	Boot *Sensitivity*	Boot *Specificity*	**CV10** *AUC*
GLM	0.985	0.973	0.952	0.987 ± 0.008	0.988 ± 0.018	0.954 ± 0.035	0.982
RF	0.9533	0.963	0.929	0.986 ± 0.011	0.987 ± 0.010	0.972 ± 0.022	0.962
LASSO	0.9854	0.995	0.929	0.987 ± 0.008	0.986 ± 0.021	0.951 ± 0.036	0.979
XGB	0.9997	0.995	1	1.000 ± 0.000	0.999 ± 0.002	1.000 ± 0.000	0.966

### SLC7A11 regulates ferroptosis resistance through NRF2-GSH-GPX4 axis

**Figure 5:**
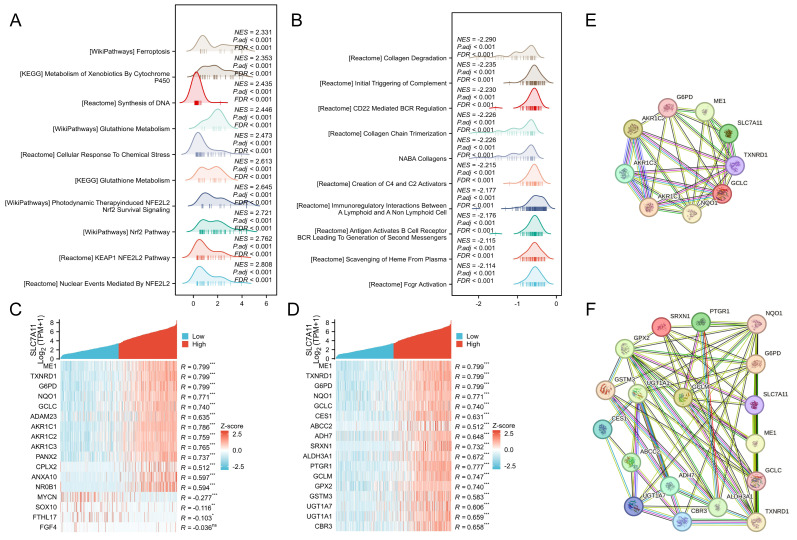
Functional enrichment and PPI analysis. (A) Positively enriched pathways in tumors with high SLC7A11 expression. (B) Negatively enriched pathways in tumors with SLC7A11 expression. (C) Heatmap of genes co-expressed with SLC7A11 that are related to ferroptosis. (D) Heatmap of genes co-expressed with SLC7A11 that are involved in NRF2 signaling. (E) PPI networks of ferroptosis-related gene sets (F) PPI networks of NRF2-related gene sets. NES, normalized enrichment score; FDR, false discovery rate.

Differential expression analyses revealed that SLC7A11-high tumors exhibit strong activation of redox homeostasis and ferroptosis resistance pathways. Consistently, GSEA showed robust enrichment of NRF2 signaling, glutathione metabolism, and ferroptosis-related programs (NES > 2.3, FDR < 0.001; Fig. 5A), suggesting that SLC7A11 overexpression might enhance oxidative stress adaptation. In contrast, immune regulatory pathways, including complement activation, B cell receptor signaling, and Fcγ receptor-mediated responses, were markedly suppressed (NES < −2.1, FDR < 0.001; Fig. 5B), indicating elevated SLC7A11 could facilitate immunosuppression.

To clarify mechanistic axis linking SLC7A11 to ferroptosis suppression, intersecting DEGs with ferroptosis-related genes (FRGs) and NRF2-related genes (NRGs) identified 17 genes in each category (Figs. 5C–5D). These included critical regulators of cystine uptake, glutathione synthesis, NADPH generation, and peroxide detoxification (*e.g.*, GCLC, GCLM, NQO1, TXNRD1, ME1, and G6PD). PPI network analysis further revealed dense interconnections among these metabolic regulators, with SLC7A11 positioned centrally within a cluster governing the NRF2-GSH-GPX4 antioxidant system (Figs. 5E–5F). These findings support SLC7A11 as a key metabolic hub linking ferroptosis resistance, redox adaptation, and immune modulation in LUSC.

### SLC7A11 reshaped the immune microenvironment in LUSC

**Figure 6:**
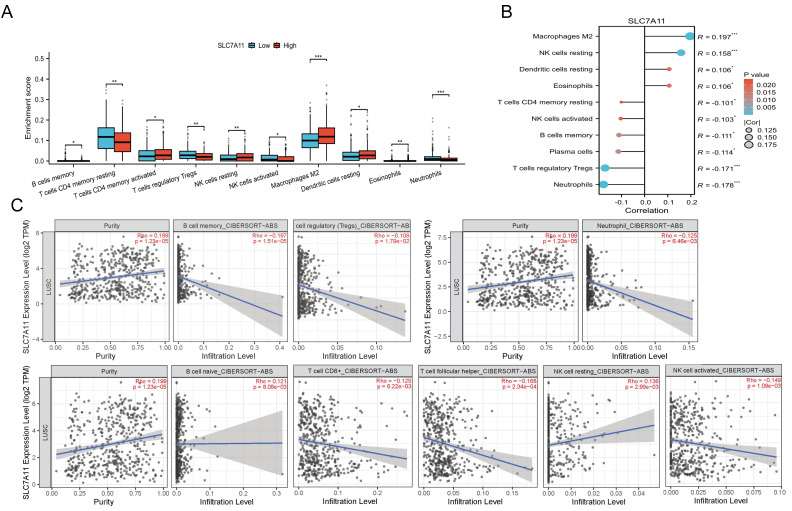
Immune infiltration associated with SLC7A11 expression in LUSC. (A) Differences in immune cell infiltration between SLC7A11-high and SLC7A11-low tumors estimated by CIBERSORT. (B) Correlation between SLC7A11 expression and immune infiltration levels. (C) Validation of immune infiltration profile by TIMER2.0. * *P* < 0.05, ** *P* < 0.01, ***P < 0.001.

To investigate whether SLC7A11 influences the immune microenvironment, we performed CIBERSORT deconvolution on TCGA-LUSC bulk RNA-seq data. SLC7A11-high tumors exhibited a distinctly immunosuppressive microenvironment, characterized by increased infiltration of M2 macrophages, resting natural killer (NK) cells, and resting dendritic cells, alongside reduced activated NK cells, regulatory T cells (Tregs), and resting memory CD4^+^ T cells (all *P* < 0.05; Figs. 6A–6B). Spearman’s correlation further confirmed these associations, showing negative correlations between SLC7A11 and cytotoxic immune subsets while positively correlating with immunosuppressive populations. To ensure result consistency, we additionally validated these trends using the independent TIMER2.0 platform (CIBERSORT-ABS absolute mode). Consistently, TIMER2.0 demonstrated increased M2 macrophages and decreased activated NK and CD4^+^ T cells in the SLC7A11-high group (Fig. 6C).

**Figure 7:**
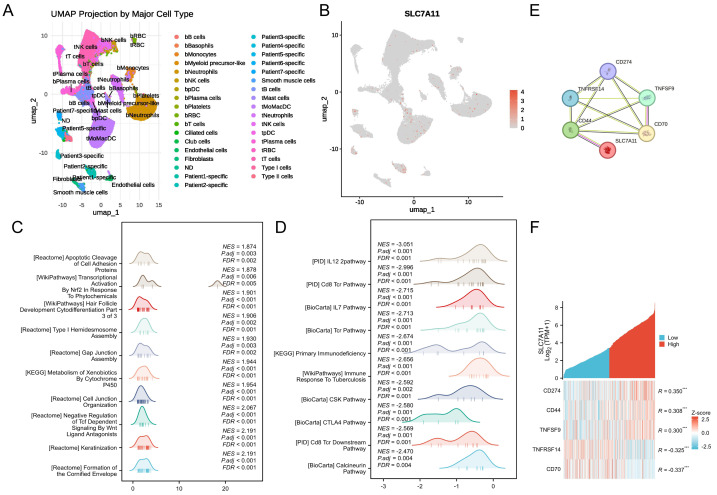
Single-cell landscape of SLC7A11 and crosstalk of SLC7A11 and immune checkpoints in LUSC. (A) UMAP project of major cell types in single-cell RNA-seq data. (B) Distribution of SLC7A11 expression across cell clusters. (C) Pathways enriched in epithelial cells with high SLC7A11 expression. (C) Pathways enriched in epithelial cells with low SLC7A11 expression. (E) PPI network of SLC7A11 and related immune-checkpoint molecules. (F) Heatmap of co-expression between SLC7A11 and immune-checkpoint genes. *** *P* < 0.001.

scRNA-seq (GSE127465) analysis was then used to further characterized the cellular origin and downstream pathways associated with SLC7A11 expression. SLC7A11 expression was mainly localized to malignant epithelial cells rather than immune subsets (Figs. 7A–7B). Pathway enrichment analysis of SLC7A11-high epithelial cells showed marked activation of oxidative stress adaptation and NRF2-related metabolic programs, together with suppression of T cell receptor (TCR) signaling, IL-12/IL-7 pathway, and CTLA-4 signaling (Figs. 7C–7D).

Immune checkpoint co-expression analysis revealed significant correlations between SLC7A11 expression and several immune checkpoint genes, most notably CD274 (PD-L1) (*R* = 0.350, *P* < 0.001), as well as CD44 and TNFSF9 (Fig. 7F). STRING network analysis further confirmed strong interconnectivity among these checkpoints (Fig. 7E).

Collectively, these finding suggest that SLC7A11 might facilitate immune evasion in LUSC through promoting immunosuppressive cell infiltration and coordinating the upregulation of immune checkpoint pathways.

### Drug sensitivity profiling highlights SLC7A11 as a predictive biomarker for therapeutic response

To determine whether SLC7A11 expression influences therapeutic vulnerability. GDSC-based pharmacogenomic analysis *via* GSCALite revealed that high SLC7A11 expression was significantly associated with increased resistance to multiple histone deacetylase (HDAC) inhibitors and reactive oxygen species (ROS), inducing agents (*e.g.*, vorinostat, piperlongumine). Conversely, high SLC7A11 expression was linked to enhanced sensitivity to specific targeted compounds, including recombinant TRAIL (rTRAIL), the HSP90 inhibitor 17-AAG, and the Ras farnesyltransferase inhibitor FTI-277 (Supplementary Materials S13, S14). Collectively, these findings suggest that SLC7A11 may serve as a predictive biomarker for therapeutic response heterogeneity and provide a potential basis for precision treatment stratification in LUSC.

## Discussion

LUSC diagnosis still relies heavily on low-dose CT screening and invasive tissue sampling, while standard chemotherapy and radiotherapy often confer limited benefit and substantial toxicity. These limitations underscore the need for minimally invasive biomarkers that improve early detection and guide precision therapy. In this study, we delineated a LUSC-specific pattern of SLC7A11epigenetic activation by integrating DNA methylation, transcriptomic, proteomic, and immune microenvironment analyses to clarify its biological and clinical significance.

DNA methylation plays a central regulatory role in lung cancer pathogenesis, with broad applications in early diagnosis, subtype classification, and prediction of immunotherapy response (Hulbert et al., 2017; Ooki et al., 2017; Kim, Choi & Jung, 2020; Peinado et al., 2022; Zeng et al., 2024; Zhang et al., 2025c). Our data revealed that SLC7A11 is uniquely activated in LUSC through coordinated promoter and gene-body hypomethylation, accompanied by marked mRNA and protein upregulation. This tumor-specific multi-omics activation was not observed across other cancer types, highlighting a distinct regulatory landscape in LUSC. Notably, this promoter hypomethylation showed no racial variation, suggesting that its alteration is predominantly tumor-intrinsic rather than ethnicity-dependent. Meanwhile, sex-associated methylation differences further suggests potential hormonal or chromatin-accessibility influences, consistent with prior evidence showing sex-biased epigenetic regulation in thoracic malignancies (Grant et al., 2022; Patel et al., 2024).

Leveraging this epigenetic signature, we developed a four-CpG methylation diagnostic classifier that achieved near-perfect accuracy in both TCGA and an external GEO validation cohort. Methylation-based markers, particularly those located in TSS-proximal regions, are known for their stability and suitability for blood-based assays (Wei et al., 2021). Our identification of cg02102889 (TSS1500) as the dominant diagnostic contributor further reinforces its translational relevance. Although prospective validation, especially in in plasma or bronchial lavage specimens, is needed, our findings emphasize the feasibility of incorporating SLC7A11 methylation markers into early screening workflows (Li et al., 2020a; Fang et al., 2022; Li et al., 2022b; Müller & Gyorffy, 2025; Trombetta et al., 2025).

Beyond diagnostic implications, our study provides biological insight into SLC7A11-driven tumor behavior. Consistent with its established role in ferroptosis suppression, SLC7A11-high tumors exhibited pronounced activation of the NRF2-GSH-GPX4 antioxidant axis (Gong et al., 2022; Su et al., 2025; Yan et al., 2025). Importantly, these metabolic changes were coupled with broad suppression of immune-regulatory pathways. Such cross-talk aligns with emerging evidence that metabolic rewiring and antioxidant programs can remodel the tumor immune microenvironment and attenuate anti-tumor immunity (Gao et al., 2025; Xu et al., 2025a). The immunosuppressive microenvironment associated with SLC7A11 overexpression has important implications for immunotherapy response. Extensive evidence shows that tumor-associated macrophages, NK-cell dysfunction and cytokine-driven signaling networks collectively shape sensitivity to immune-checkpoint blockade. Recent studies further highlight how epigenetic regulators and metabolic stress pathways remodel the TME and modulate immunotherapy efficacy (Zhu et al., 2023; Wen et al., 2024). These findings support the notion that SLC7A11-mediated metabolic reprogramming may not only promote ferroptosis resistance but also create an immune contexture less permissive to effective anti-tumor immunity (Lin et al., 2025).

Therapeutically, SLC7A11 has been implicated in resistance to oxidative stress-based chemotherapies. Our pharmacogenomic analysis is consistent with this concept, revealing increased resistance to HDAC inhibitors and ROS-inducing agents in tumors with high SLC7A11 expression. Conversely, increased sensitivity to pro-apoptotic agents such as rTRAIL and 17-AAG suggests potential therapeutic vulnerabilities. These observations parallel prior evidence that ferroptosis resistance alters susceptibility to apoptosis-based strategies (Hu et al., 2022; Ye et al., 2024; Zhuang et al., 2025). Although *in vitro* IC50 correlations require validation in patient-derived systems, these findings highlight SLC7A11 expression as a potential stratification marker to optimize treatment choice and exploit ferroptosis-related vulnerabilities in LUSC.

This study has limitations. Mechanistic conclusions were primarily based on computational analyses and require experimental validation. Prospective studies assessing the performance of the four CpG markers in plasma or bronchial lavage samples are essential to establish clinical utility. Additionally, drug sensitivity predictions should be confirmed using *in vitro* or *in vivo* LUSC models. Moreover, despite the availability of TCGA and CPTAC, these resources do not provide matched methylation, transcriptomic, and proteomic profiles from the same LUSC patients. Consequently, future studies should prospectively collect LUSC patient samples with fully matched multi-omics profiling to allow definitive characterization of the methylation-mRNA-protein regulatory axis of SLC7A11.

## Conclusion

This study identifies SLC7A11 as a LUSC-specific biomarker driven by promoter hypomethylation. A four-probe methylation signature demonstrated near-perfect diagnostic performance, indicating its potential for future blood-based screening applications. Mechanistic and drug-sensitivity analyses further suggest that SLC7A11 may inform therapeutic stratification. Overall, SLC7A11 offers a clinically meaningful avenue for improving early diagnosis and personalized treatment in LUSC.

##  Supplemental Information

10.7717/peerj.20686/supp-1 Supplemental Information 1 SLC7A11 expression across pan-cancer datasets (A) Differential expression a nalysis in 23 paired tumor and normal tissues from TCGA. (B) Expression across 34 cell lines in CCLE.

10.7717/peerj.20686/supp-2 Supplemental Information 2 Promoter methylation and expression analysis of SLC7A11 in multiple cancers using TCGA and CPTAC datasets Promoter methylation levels and mRNA expression levels of SCL7A11 in: (A-B) colon adenocarcinoma (COAD), (C-D) cervical squamous cell carcinoma (CESC), (E-F) kidney renal papillary cell carcinoma (KIRP), (G-H) kidney renal clear cell carcinoma (KIRC), (I-J) hepatocellular carcinoma (LIHC), (K-L) lung adenocarcinoma (LUAD), (M-N) pancreatic adenocarcinoma (PAAD), (O-P) rectal adenocarcinoma (READ), (Q-R) sarcoma (SARC). Protein expression levels of SLC7A11 in: (S) head and neck squamous cell carcinoma, (T) glioblastoma multiforme.

10.7717/peerj.20686/supp-3 Supplemental Information 3 Diagnostic ROC analysis of SLC7A11 across pan-cancer

10.7717/peerj.20686/supp-4 Supplemental Information 4 Pan-cancer survival analysis based on SLC7A11 expression

10.7717/peerj.20686/supp-5 Supplemental Information 5 Association bet ween SLC7A11 expression and clinical variables across pan-cancer cohort

10.7717/peerj.20686/supp-6 Supplemental Information 6 Survival analysis of SLC7A11 in LUSC (A) Overall survival (OS) stratified by SLC7A11 expression. (B) Disease-specific survival (DSS) according to SLC7A11 expression. (C) Progression-free interval (PFI) in high vs. low SLC7A11 groups. (D) PFI analysis in female patients stratified by SLC7A11 expression. (E–F) OS (E) and PFI (F) in patients with advanced pathologic T stage (T3 & T4). (G–H) Survival analysis based on CpG methylation sites cg05373863 (G) and cg24869834 (H). HR: hazard ratio; P values from log-rank tests.

10.7717/peerj.20686/supp-7 Supplemental Information 7 Raw IHC data for SLC7A1 from the in-house cohort (173 paired tumor and normal tissues)

10.7717/peerj.20686/supp-8 Supplemental Information 8 Protein expression of SLC7A11 ac ross clin ic opathogical parameters in in-house cohort (A) Age , (B) pT stage , (C) pN stage , (D) pM stage , (E) pStage , (F) Ki-67 level.

10.7717/peerj.20686/supp-9 Supplemental Information 9 Promoter methylation levels of SLC7A11 across different racial groups in TCGA-LUSC Comparison of *β* -v alues among normal lung tissues, Caucasian, African American, and Asian LUSC samples. ns, not significant; *P ¡ 0.05; ***P ¡ 0.001.

10.7717/peerj.20686/supp-10 Supplemental Information 10 M icroarrays and RNA-Seq datasets for SLC7A11 mRNA analysis us ed in this study

10.7717/peerj.20686/supp-11 Supplemental Information 11 Analysis codes for SLC7A11 methylation profiling and scRNA-seq interrogation in LUSC

10.7717/peerj.20686/supp-12 Supplemental Information 12 Correlation betw een SLC7A11 expression and clinicopathological parameters in TCGA and in-house cohort s

10.7717/peerj.20686/supp-13 Supplemental Information 13 Drug sensitivity analysis of SLC7A11

10.7717/peerj.20686/supp-14 Supplemental Information 14 Detailed c orrelation between GDSC drug sensitivity and SLC7A11 expression Comprehensive drug-gene correlation data obtained from GSCA Lite (https://guolab.wchscu.cn/GSCA/#/drug) .

10.7717/peerj.20686/supp-15 Supplemental Information 15 TCGA clinical data

## Abbreviations

5′-UTR: 5′ untranslated region
AUC: Area under the curve
CPTAC: Clinical Proteomic Tumor Analysis Consortium
DEG: Differentially expressed genes
DMP: Differentially methylated probes
DSS: Disease-specific survival
FDR: False discovery rate
FRGs: Ferroptosis-related genes
GDSC: Genomics of Drug Sensitivity in Cancer
GEO: Gene Expression Omnibus
GLM: Generalized linear model
GPX4: Glutathione peroxidase 4
GSEA: Gene set enrichment analysis
GSH: Glutathione
HDAC: Histone deacetylase
IC50: Half-maximal inhibitory concentration
IHC: Immunohistochemistry
LASSO: Least absolute shrinkage and selection operator
LUSC: Lung squamous cell carcinoma
NRF2: Nuclear factor erythroid 2-related factor 2
NRGs: NRF2-related genes
NSCLC: Non-small cell lung cancer
OS: Overall survival
PCA: Principal component analysis
PD-L1: Programmed death-ligand 1
PFI: Progression-free interval
PPI: Protein-protein interaction
RF: Random forest
ROC: Receiver operating characteristic
ROS: Reactive oxygen species
rTRAIL: recombinant TRAIL
SHAP: Shapley additive explanations
SLC7A11: Solute carrier family 7 member 11
TCGA: The Cancer Genome Atlas
TCR: T cell receptor
TME: Tumor microenvironment
Treg: Regulatory T cell
TSS: transcription start site
UMAP: Uniform Manifold Approcimation and Projection
XGB: Extreme gradient boosting

## Additional Information and Declarations

### DNA Deposition

The following information was supplied regarding the deposition of DNA sequences: The data is available at TCGA, GEO (GSE121849, GSE127465) and CPTAC.

### Data Availability

The following information was supplied regarding data availability: The raw data is available in the Supplementary Files. The data is available at TCGA, GEO (GSE121849, GSE127465) and CPTAC.
