# Supplementary figures and images for: Epigenetic activation of SLC7A11 defines a ferroptosis—immune axis and enables robust DNA methylation-based diagnosis of lung squamous cell carcinoma
Source: PeerJ. 2026 Feb 12;14:e20686. doi: 10.7717/peerj.20686 (PMC12906708; doi:10.7717/peerj.20686)

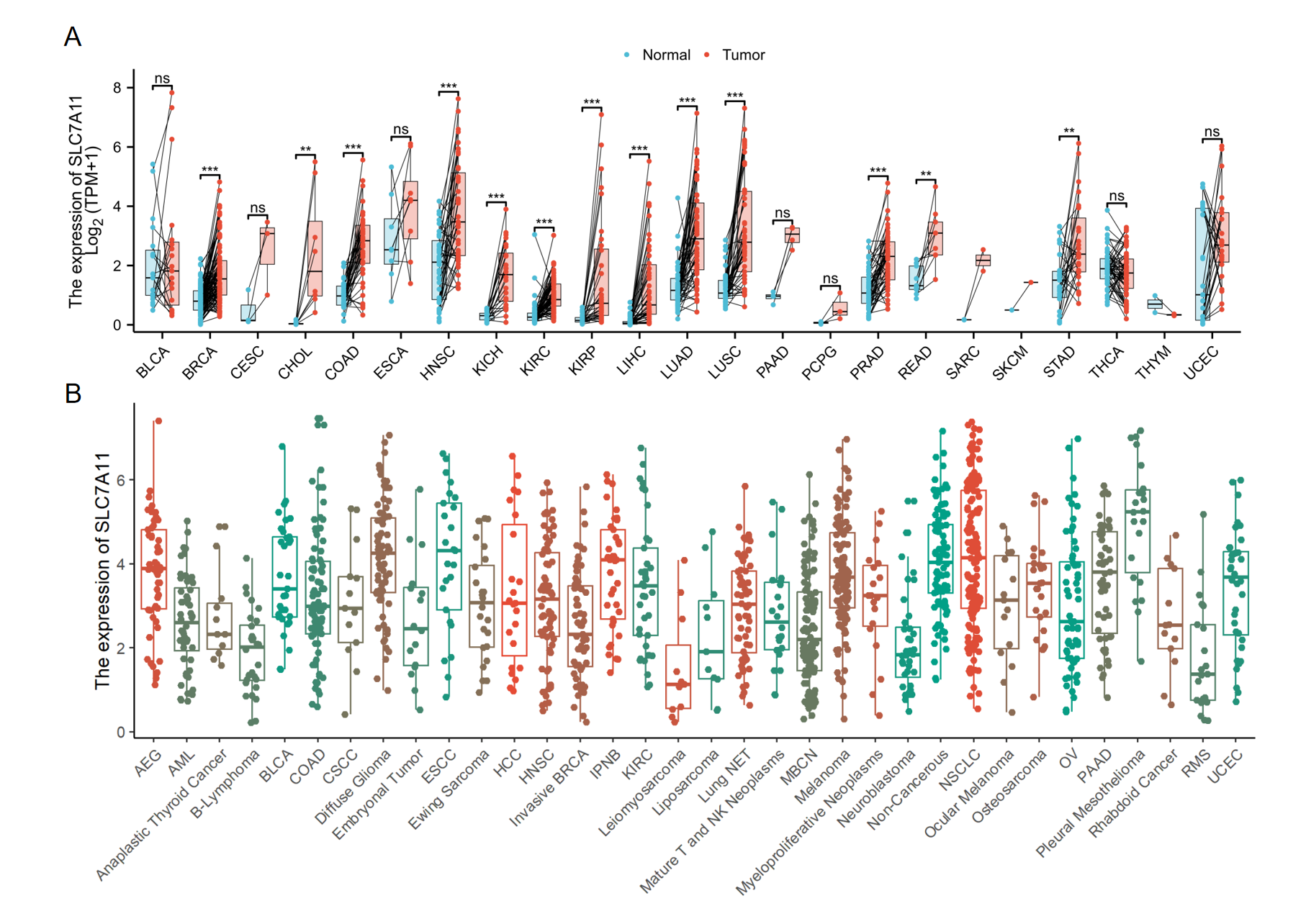

Supplement: Supplemental Information 1 — (A) Differential expression a nalysis in 23 paired tumor and normal tissues from TCGA. (B) Expression across 34 cell lines in CCLE. [file peerj-14-20686-s001.png]

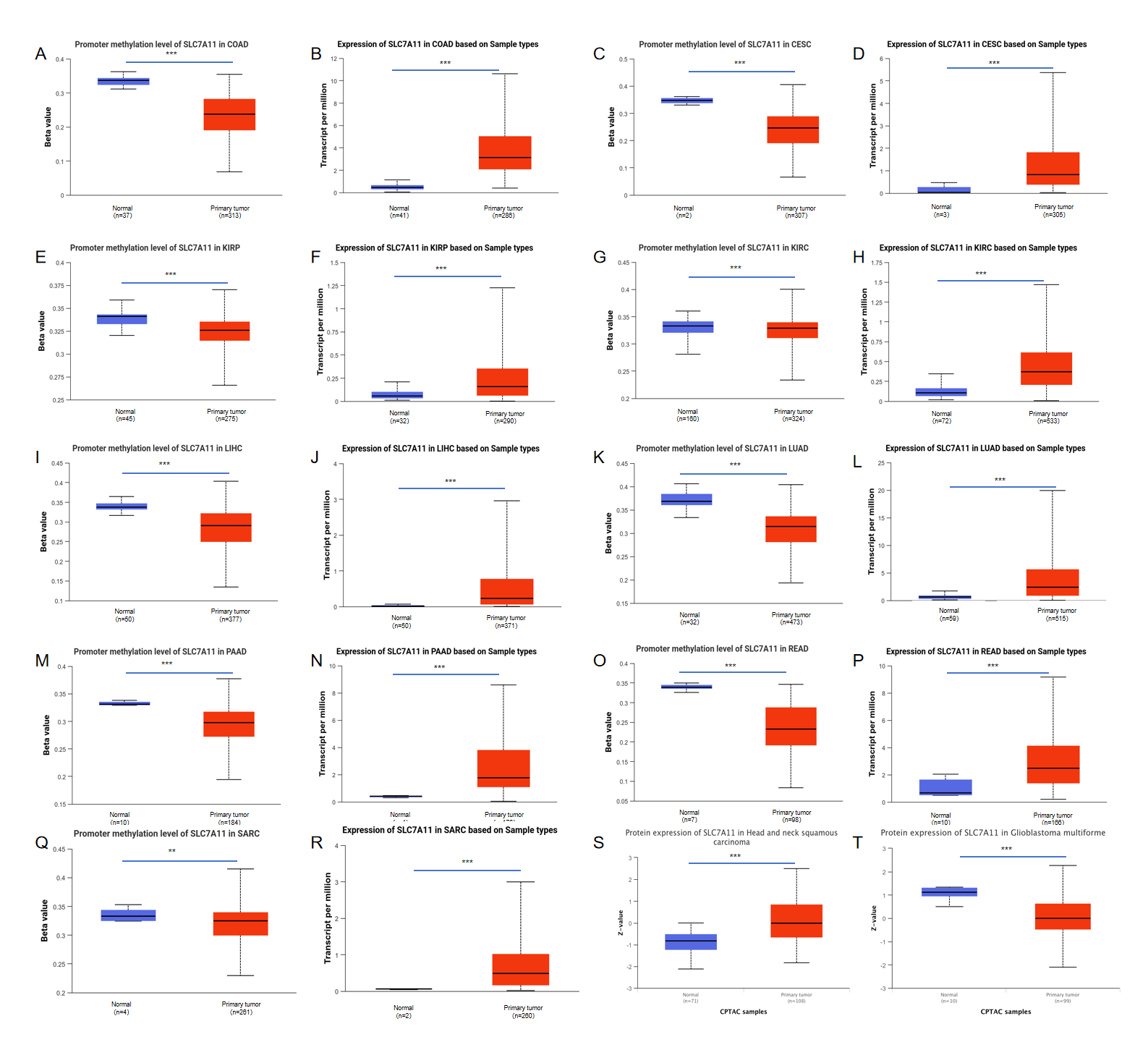

Supplement: Supplemental Information 2 — Promoter methylation levels and mRNA expression levels of SCL7A11 in: (A-B) colon adenocarcinoma (COAD), (C-D) cervical squamous cell carcinoma (CESC), (E-F) kidney renal papillary cell carcinoma (KIRP), (G-H) kidney renal clear cell carcinoma (KIRC), (I-J) hepatocellular carcinoma (LIHC), (K-L) lung adenocarcinoma (LUAD), (M-N) pancreatic adenocarcinoma (PAAD), (O-P) rectal adenocarcinoma (READ), (Q-R) sarcoma (SARC). Protein expression levels of SLC7A11 in: (S) head and neck squamous cell carcinoma, (T) glioblastoma multiforme. [file peerj-14-20686-s002.png]

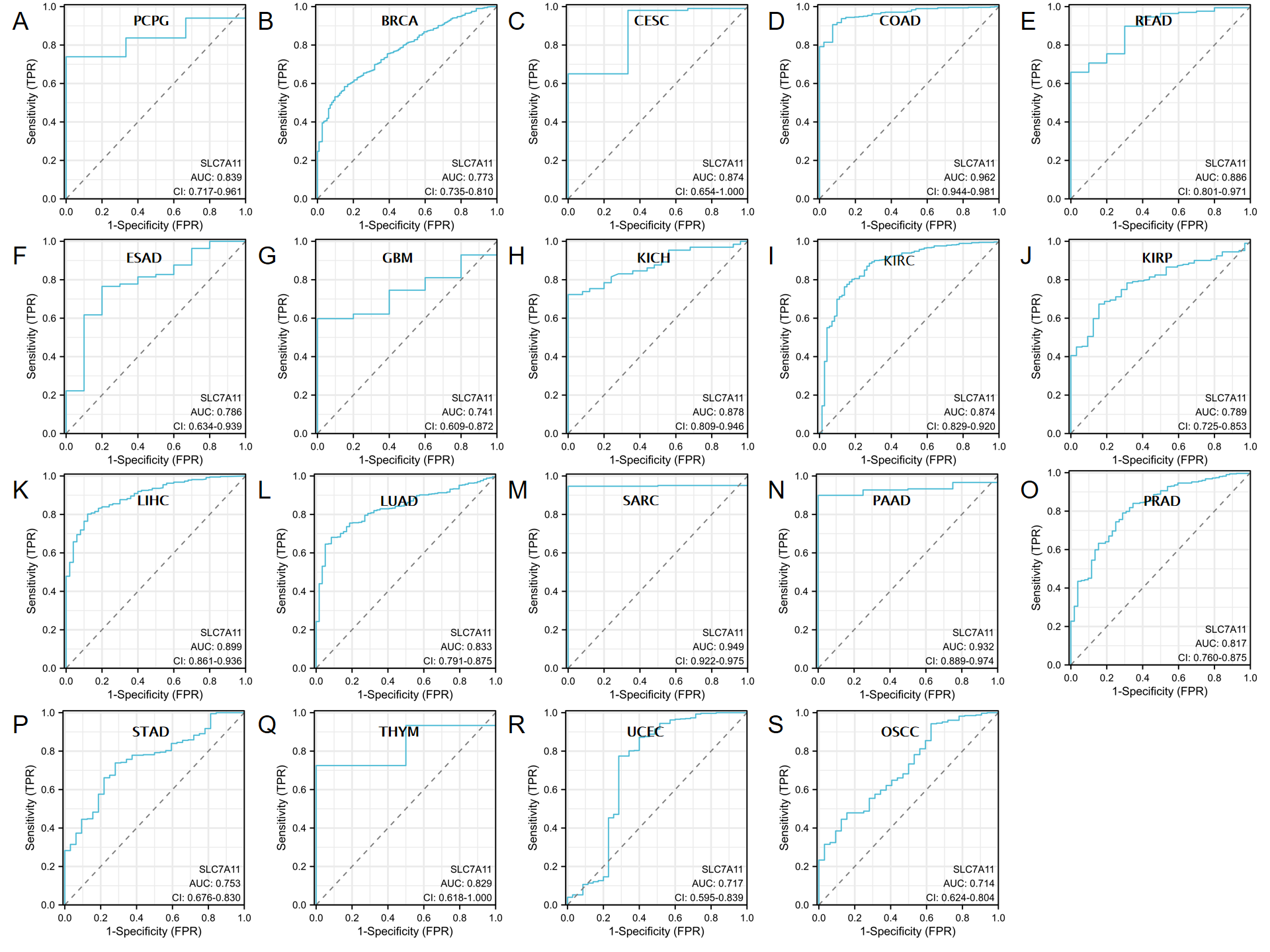

Supplement: Supplemental Information 3 [file peerj-14-20686-s003.png]

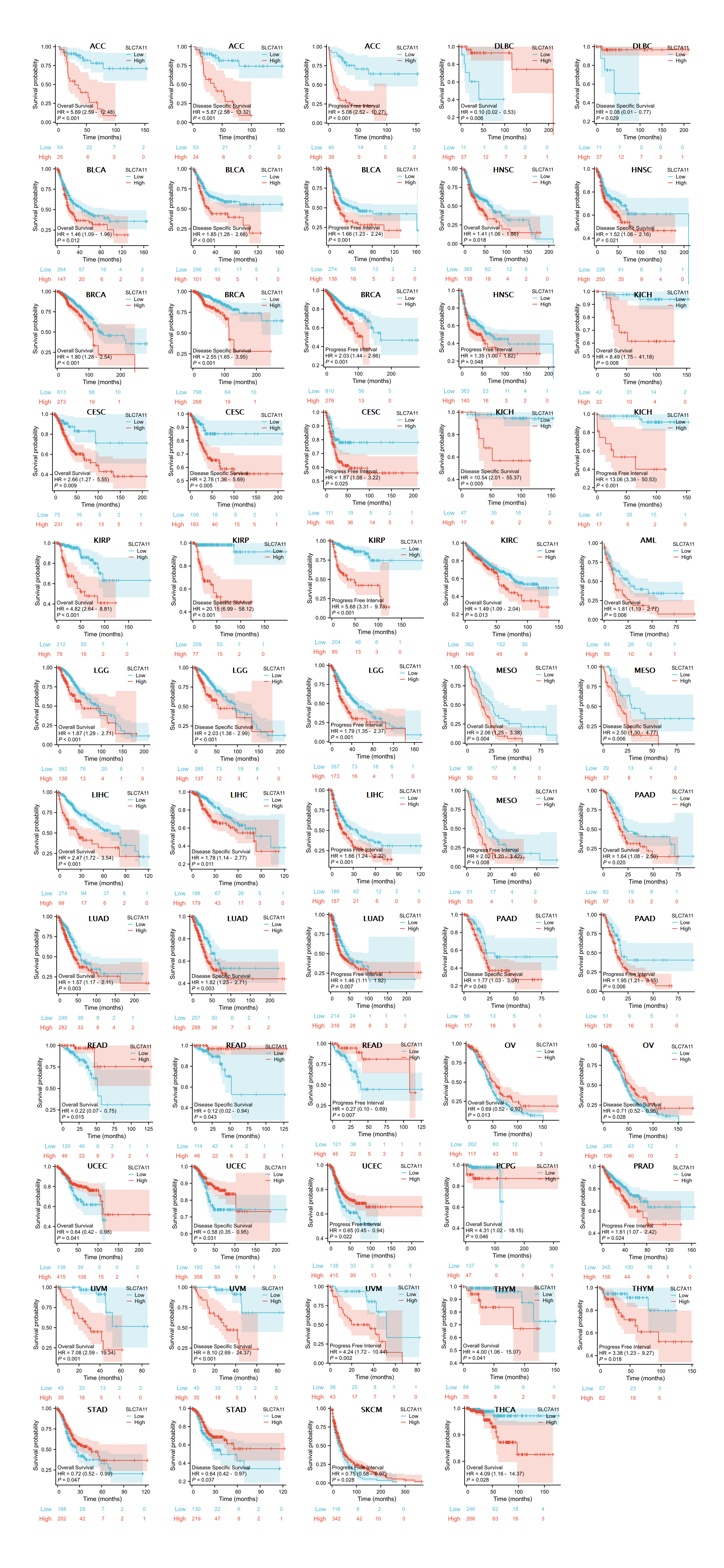

Supplement: Supplemental Information 4 [file peerj-14-20686-s004.png]

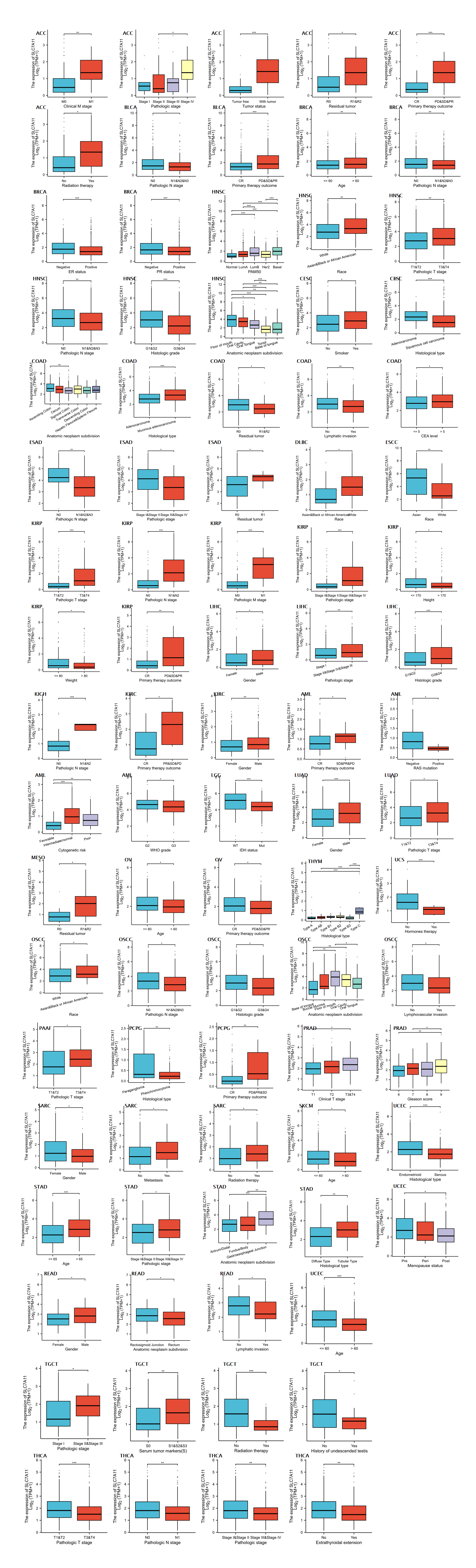

Supplement: Supplemental Information 5 [file peerj-14-20686-s005.png]

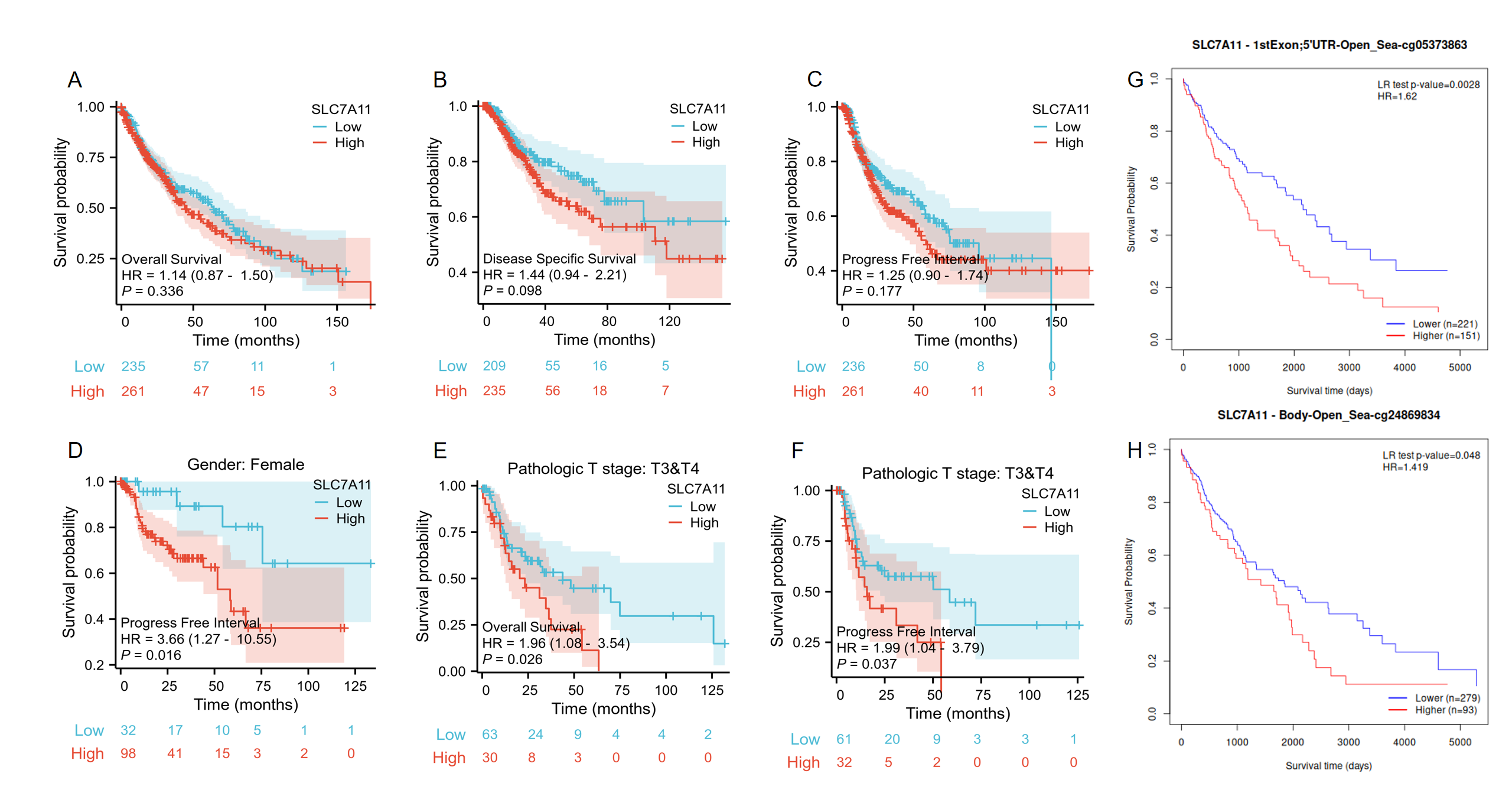

Supplement: Supplemental Information 6 — (A) Overall survival (OS) stratified by SLC7A11 expression. (B) Disease-specific survival (DSS) according to SLC7A11 expression. (C) Progression-free interval (PFI) in high vs. low SLC7A11 groups. (D) PFI analysis in female patients stratified by SLC7A11 expression. (E–F) OS (E) and PFI (F) in patients with advanced pathologic T stage (T3 & T4). (G–H) Survival analysis based on CpG methylation sites cg05373863 (G) and cg24869834 (H). HR: hazard ratio; P values from log-rank tests. [file peerj-14-20686-s006.png]

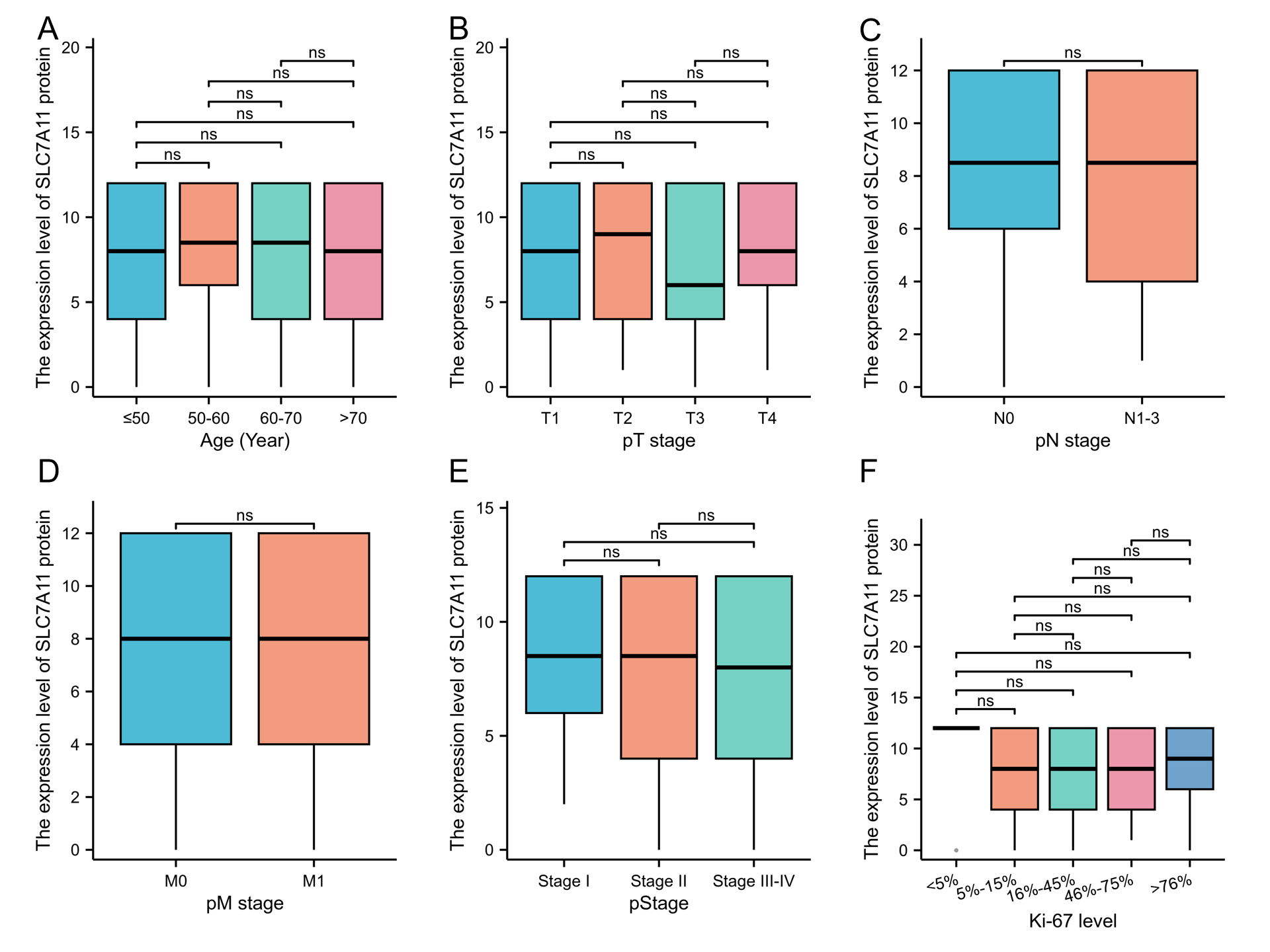

Supplement: Supplemental Information 8 — (A) Age , (B) pT stage , (C) pN stage , (D) pM stage , (E) pStage , (F) Ki-67 level. [file peerj-14-20686-s008.png]

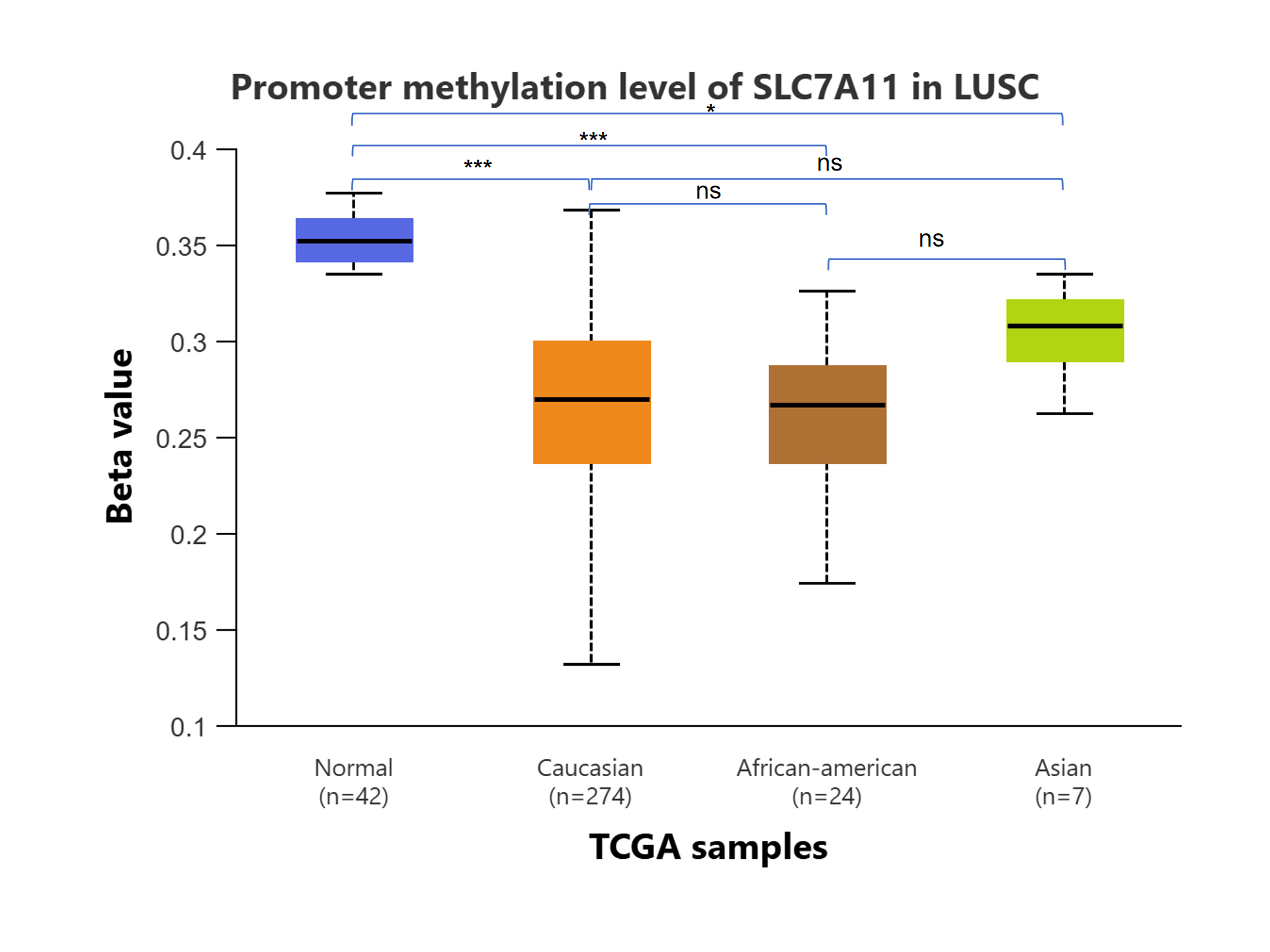

Supplement: Supplemental Information 9 — Comparison of β -v alues among normal lung tissues, Caucasian, African American, and Asian LUSC samples. ns, not significant; *P ¡ 0.05; ***P ¡ 0.001. [file peerj-14-20686-s009.png]

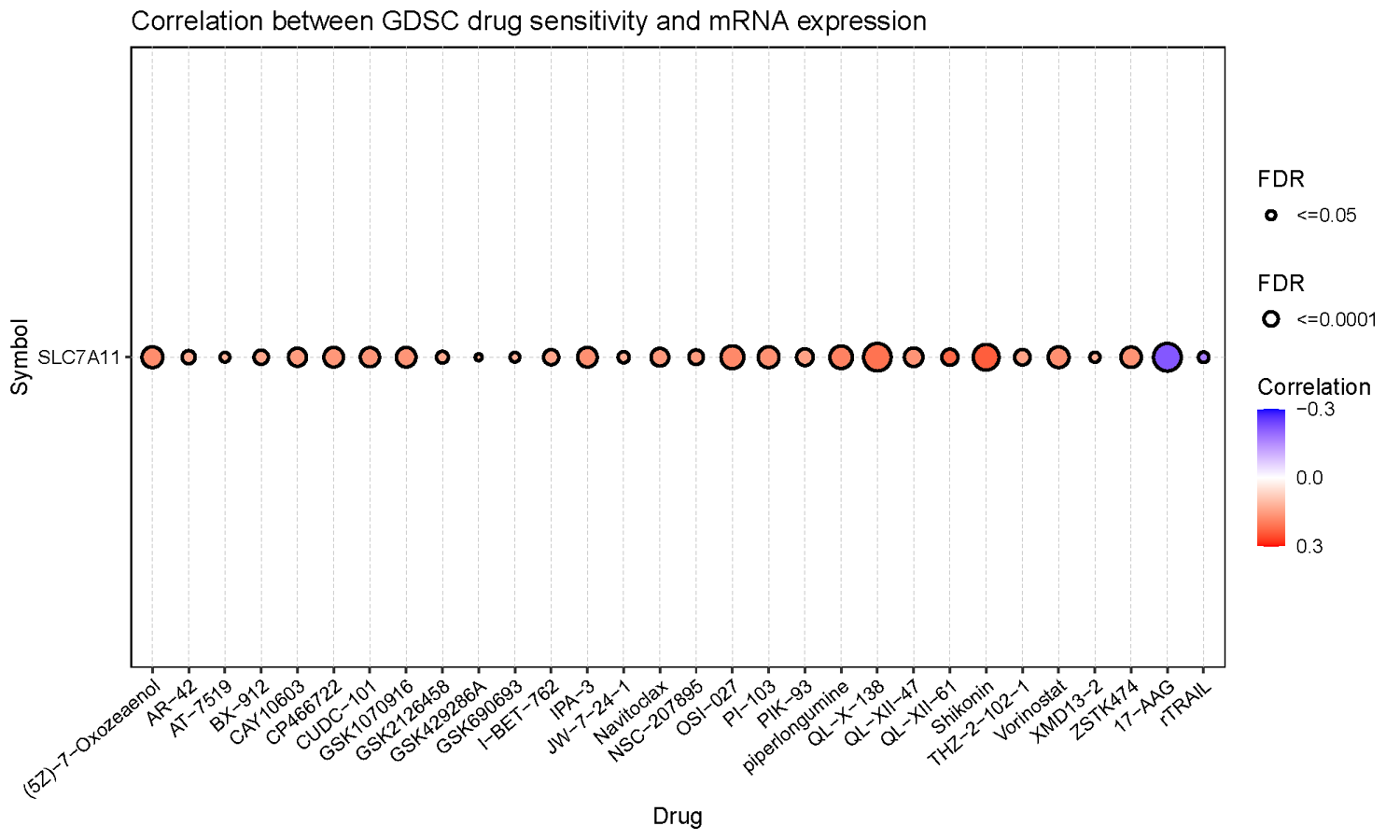

Supplement: Supplemental Information 13 [file peerj-14-20686-s013.png]
