# Supplementary material for: Epigenetic activation of SLC7A11 defines a ferroptosis—immune axis and enables robust DNA methylation-based diagnosis of lung squamous cell carcinoma
Source: PeerJ. 2026 Feb 12;14:e20686. doi: 10.7717/peerj.20686 (PMC12906708; doi:10.7717/peerj.20686)
Supplement: Supplemental Information 10 [file peerj-14-20686-s010.docx]

**Supplementary material 10.** SLC7A11 mRNA-related microarrays and RNA-Seq datasets included in this study.

| Platform | Dataset | LUSC sample | Non-LUSC sample |
| --- | --- | --- | --- |
| GPL13534 | GSE121849 | 20 | 13 |
| GPL18573 | GSE127465 | 7 | 0 |
|  | TCGA | 502 | 49 |
