# Supplementary material for: Epigenetic activation of SLC7A11 defines a ferroptosis—immune axis and enables robust DNA methylation-based diagnosis of lung squamous cell carcinoma
Source: PeerJ. 2026 Feb 12;14:e20686. doi: 10.7717/peerj.20686 (PMC12906708; doi:10.7717/peerj.20686)
Supplement: Supplemental Information 12 [file peerj-14-20686-s012.docx]

1. The association between SLC7A11 expression and clinical pathological parameters in the TCGA-LUSC cohort.

| **Characteristics** | **SLC7A11-Low** | **SLC7A11-High** | ***P* value** |
| --- | --- | --- | --- |
| n | 251 | 251 |  |
| **Gender, n (%)** |  |  | **0.019** |
| Female | 77 (15.3%) | 54 (10.8%) |  |
| Male | 174 (34.7%) | 197 (39.2%) |  |
| Race, n (%) |  |  | 0.408 |
| Asian&Black or African American | 17 (3.4%) | 22 (4.4%) |  |
| White | 177 (35.3%) | 173 (34.5%) |  |
| Unknown | 57 (11.4%) | 56 (11.1%) |  |
| Age, n (%) |  |  | 0.099 |
| <= 65 | 86 (17.1%) | 105 (20.9%) |  |
| > 65 | 159 (31.7%) | 143 (28.5%) |  |
| Unknown | 6 (1.2%) | 3 (0.6%) |  |
| Pathologic T stage, n (%) |  |  | 0.184 |
| T1 | 65 (12.9%) | 49 (9.8%) |  |
| T2 | 138 (27.5%) | 156 (31.1%) |  |
| T3&T4 | 48 (9.6%) | 46 (9.2%) |  |
| Pathologic N stage, n (%) |  |  | 0.695 |
| N0 | 163 (32.5%) | 157 (31.3%) |  |
| N1 | 64 (12.7%) | 67 (13.3%) |  |
| N2&N3 | 20 (4.0%) | 25 (5.0%) |  |
| Unknown | 4 (0.8%) | 2 (0.4%) |  |
| Pathologic M stage, n (%) |  |  | 0.481 |
| M0 | 209 (41.6%) | 203 (40.4%) |  |
| M1 | 5 (1.0%) | 2 (0.4%) |  |
| Unknown | 37 (7.4%) | 46 (9.2%) |  |
| Pathologic stage, n (%) |  |  | 0.949 |
| Stage I | 125 (24.9%) | 120 (23.9%) |  |
| Stage II | 80 (15.9%) | 82 (16.3%) |  |
| Stage III&Stage IV | 46 (9.2%) | 45 (9.0%) |  |
| Unknown | 0 (0.0%) | 4 (0.8%) |  |
| Residual tumor, n (%) |  |  | 0.285 |
| R0 | 204 (40.6%) | 195 (38.8%) |  |
| R1&R2 | 6 (1.2%) | 10 (2.0%) |  |
| Unknown | 41 (8.2%) | 46 (9.2%) |  |
| Anatomic neoplasm subdivision, n (%) |  |  | 0.497 |
| Left | 110 (21.9%) | 104 (20.7%) |  |
| Right | 125 (24.9%) | 134 (26.7%) |  |
| Unknown | 16 (3.2%) | 13 (2.6%) |  |
| Location, n (%) |  |  | 0.536 |
| Central Lung | 73 (14.5%) | 74 (14.7%) |  |
| Peripheral Lung | 50 (10.0%) | 43 (8.6%) |  |
| Unknown | 128 (25.5%) | 134 (26.7%) |  |
| Smoker, n (%) |  |  | 0.346 |
| No | 7 (1.4%) | 11 (2.2%) |  |
| Yes | 237 (47.2%) | 235 (46.8%) |  |
| Unknown | 7 (1.4%) | 5 (1.0%) |  |
| Number pack years smoked, n (%) |  |  | 0.468 |
| < 40 | 64 (12.7%) | 70 (13.9%) |  |
| >= 40 | 150 (29.9%) | 141 (28.1%) |  |
| Unknown | 37 (7.4%) | 40 (8.0%) |  |
| Primary therapy outcome, n (%) |  |  | 0.304 |
| CR | 163 (32.5%) | 145 (28.9%) |  |
| PD&SD&PR | 24 (4.8%) | 29 (5.8%) |  |
| Unknown | 64 (12.7%) | 77 (15.3%) |  |
| **CD274, n (%)** |  |  | **<0.001** |
| Low | 160 (31.9%) | 91 (18.1%) |  |
| High | 91 (18.1%) | 160 (31.9%) |  |

Notes: Percentages were calculated using the total number of cases in each SLC7A11 group as the denominator; missing values are shown as “Unknown”.

1. The association between SLC7A11 expression and clinical pathological parameters in the in-house LUSC cohort.

| **Characteristics** | **SLC7A11-Low** | **SLC7A11-High** | ***P* value** |
| --- | --- | --- | --- |
| n | 66 | 107 |  |
| Gender, n (%) |  |  | 0.067 |
| Female | 15 (8.7%) | 13 (7.5%) |  |
| Male | 51 (29.5%) | 94 (54.3%) |  |
| Age, mean ± sd | 60.167 ± 9.9994 | 61.131 ± 8.919 | 0.511 |
| Pathologic T stage, n (%) |  |  | 0.272 |
| T2 | 17 (10%) | 33 (19.4%) |  |
| T4 | 9 (5.3%) | 13 (7.6%) |  |
| T1 | 23 (13.5%) | 45 (26.5%) |  |
| T3 | 16 (9.4%) | 14 (8.2%) |  |
| Pathologic N stage, n (%) |  |  | 0.240 |
| N1-3 | 20 (12.3%) | 26 (16%) |  |
| N0 | 39 (24.1%) | 77 (47.5%) |  |
| Pathologic M stage, n (%) |  |  | 0.913 |
| M0 | 48 (27.7%) | 77 (44.5%) |  |
| M1 | 18 (10.4%) | 30 (17.3%) |  |
| Pathologic stage, n (%) |  |  | 0.695 |
| Stage I | 9 (5.6%) | 19 (11.9%) |  |
| Stage III-IV | 33 (20.6%) | 47 (29.4%) |  |
| Stage II | 20 (12.5%) | 32 (20%) |  |
| Ki-67, median (IQR) | 0.5 (0.4, 0.7) | 0.6 (0.4, 0.7) | 0.699 |

Notes: Continuous variables were compared using the Student’s t-test or Wilcoxon rank-sum test, as appropriate. Categorical variables were compared using the chi-square test or Fisher’s exact test. All analyses were performed using cases with available data for the corresponding variable.
